# Systematic analysis of the use of amphipathic polymers for studies of outer membrane proteins using mass spectrometry

**DOI:** 10.1016/j.ijms.2015.06.017

**Published:** 2015-11-30

**Authors:** Thomas G. Watkinson, Antonio N. Calabrese, Fabrice Giusti, Manuela Zoonens, Sheena E. Radford, Alison E. Ashcroft

**Affiliations:** a Astbury Centre for Structural Molecular Biology, School of Molecular & Cellular Biology, University of Leeds, Leeds, LS2 9JT, UK; b Laboratoire de Physico-Chimie Moléculaire des Protéines Membranaires, UMR 7099, Institut de Biologie Physico-Chimique (FRC 550), Centre National de la Recherche Scientifique/Université Paris-7, 13, rue Pierre-et-Marie-Curie, 75005 Paris, France

**Keywords:** Outer membrane proteins, Amphipols, Protein conformation, Electrospray ionisation–mass spectrometry, Ion mobility spectrometry–mass spectrometry

## Abstract

Membrane proteins (MPs) are essential for numerous important biological processes. Recently, mass spectrometry (MS), coupled with an array of related techniques, has been used to probe the structural properties of MPs and their complexes. Typically, detergent micelles have been employed for delivering MPs into the gas-phase, but these complexes have intrinsic properties that can limit the utility of structural studies of MPs using MS methods. Amphipols (APols) have advantages over detergent micelles and have been shown to be capable of delivering native MPs into the gas-phase. Comparing six different APols which vary in mass and charge, and the detergent *n*-dodecyl-β-d-maltopyranoside, we aimed to determine which APols are most efficient for delivery of native outer membrane proteins (OMPs) into the gas-phase. We show that maintaining the solution-phase folding and global structures of three different OMPs (PagP, OmpT and tOmpA) are independent of the APol used, but differences in OMP activity can result from the different APol:OMP complexes. ESI-IMS–MS analysis of OMP:APol complexes shows that the A8-35 APol is most proficient at liberating all three OMPs into the gas-phase, without altering their gas-phase conformations.

## Highlights

• Outer membrane protein conformational analysis.
• Use of amphipols for mass spectrometry analyses.
• Comparison of a range of amphipols for membrane protein analysis.

## Graphical abstract

**Figure:**
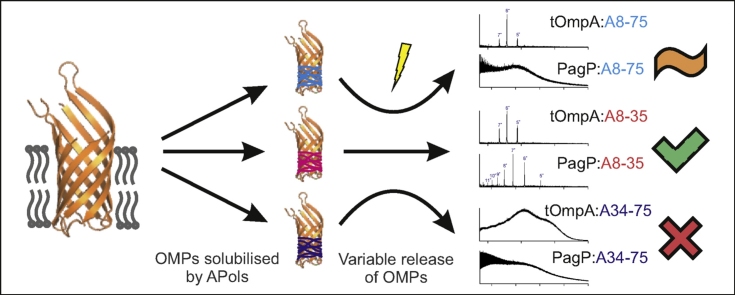

## 1 Introduction

Membrane proteins (MPs) are vital components of many biological systems and represent a proportion of drug targets greater than their abundance in the genome [1]. Despite this, high resolution structural data from conventional biophysical techniques such as X-ray crystallography and NMR spectroscopy are sparse when compared with those available for soluble proteins. This is a result of the poor aqueous solubility of MPs and the difficulty in expressing and purifying MPs in yields required for structural analysis [2]. Mass spectrometry (MS) has recently been used to probe the topology, stability and stoichiometry of MPs and their complexes, to assess specific binding of lipids and detergents to MPs, and to determine the influence of these solubilising partners on the gas-phase stability of MPs [3], [4], [5], [6], [7]. MS has also been used in combination with other techniques, such as ion mobility spectrometry (IMS), photo-oxidative labelling (FPOP) and hydrogen–deuterium exchange (HDX), to provide further structural and dynamic information of MPs in the solution and gas-phase [8], [9], [10].

**Fig. 1:**
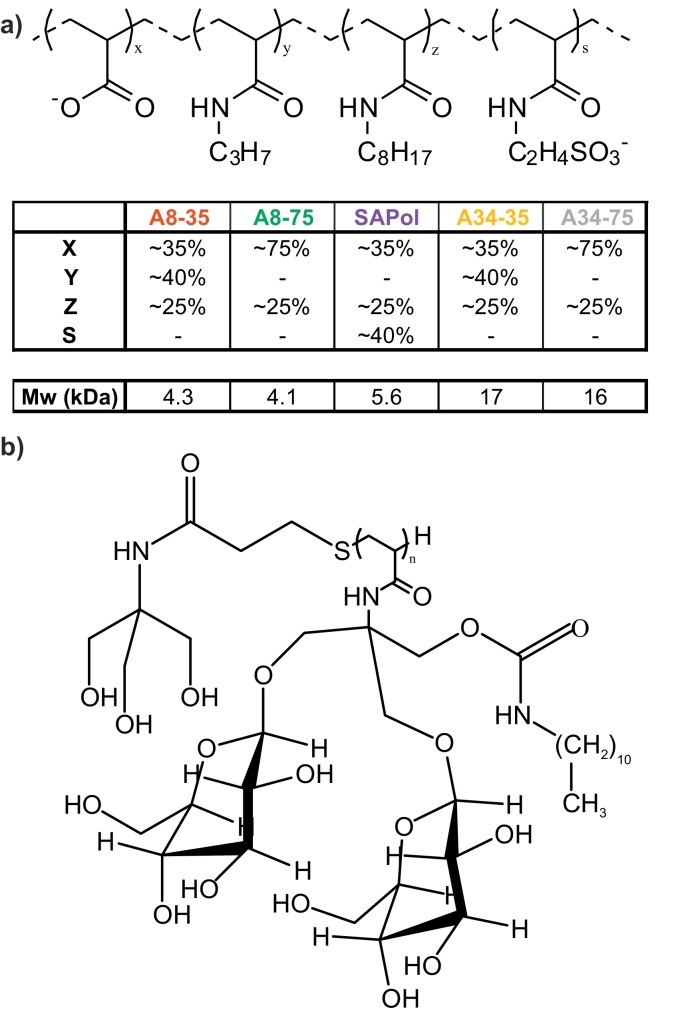
(a) Average compositions of A8-35, A8-75, SAPol, A34-35 and A34-75. The table shows the % of free acid groups that remain ungrafted (x) or grafted with isopropylamine (y), octylamine (z) or taurine (s). Also shown are (approximate) number average masses of the respective APol [19]. (b) Structure of NAPol, a non-ionic amphipol (number average mass 11.3 kDa) [29].

Detergents above their critical micelle concentration (CMC) form water soluble micelles, and these are commonly used to maintain the native structure of MPs [11], [12]. The dynamic and curved nature of micelles, however, can perturb MP structure and dynamics, and high concentrations of detergents are required to maintain MP solubility [11], [12], [13]; additionally the dissociating character of detergents can bring about deactivation of membrane proteins [14]. Alternative solubilisation techniques have been developed to provide a more stable and native-like environment, including bicelles [12], [15], [16], nanodiscs [17] and amphipols (APols) [14], [18], [19], all of which have been used for the analysis of MPs by electrospray ionisation-mass spectrometry (ESI-MS) [20], [21]. Bicelles and nanodiscs have been developed to provide an environment more closely resembling that of a native membrane, whilst APols (or amphipathic polymers) are highly non-native yet provide a very stabilising environment for MPs [14], [18]. A8-35 (Fig. 1) is the best characterised of previously described APols [14], [18], [21], [22], [23]. It is a polyacrylic acid derivative randomly grafted with octylamine (∼25%) and isopropylamine (∼40%) groups for increased hydrophobicity, whilst the remaining free acid groups (∼35%) allow the solubility of APols with trapped MPs. However, as A8-35 is dependent on having ionised carboxylic acid groups for solubility, its usage is limited to pH >7 [18], [23], [24], [25]. This has inspired the development of a variety of APols to expand the range of possible applications. Non-ionic amphipols (NAPols), sulphonated amphipols (SAPols) and phosphocholine based amphipols (PCAPols) use alternative chemical groups in place of, or in addition to, carboxylic acids to maintain solubility, and alleviate the pH restriction [14], [18], [26], [27], [28], [29], [30]. Synthesis of these and other variant forms of A8-35 has expanded the repertoire of physical properties of APols (Fig. 1). For example, A8-75 is a variant of A8-35 that uses the same precursor polyacrylic acid but lacks the isopropylamine grafting, resulting in a higher proportion of free acid groups (∼75%); A34-35 and A34-75 are equivalent in their grafting to A8-35 and A8-75, respectively, but use a larger polyacrylic acid precursor [31]. The broad variability and the resulting properties of APols allow them to be applied in conjunction with an array of biophysical techniques for MP structural and function studies, including SEC, SAXS, EM and NMR [18], [19], [32], [33], [34], [35], [36], [37], [38], [39].

**Fig. 2:**
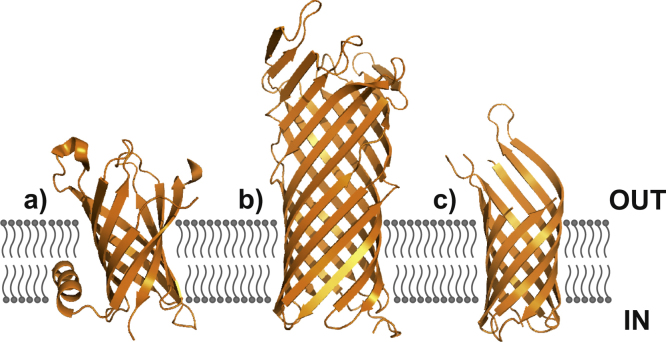
Crystal structures of (a) PagP (PDB file 1THQ) [45], (b) OmpT (PDB file 1I78) [42] and (c) tOmpA (PDB file 1QJP) [44].

Matrix-assisted laser desorption ionisation (MALDI)-MS analysis has been used for mass measurements of bacteriorhodopsin and the cytochrome *b*_*6*_*f* MP complex following release from NAPol and A8-35, respectively [35], [40]. However, due to the denaturing nature of MALDI ionisation, few conclusions can be drawn about the gas-phase structures of these MPs. Conversely, ESI-IMS–MS showed OmpT and PagP to populate a native-like conformation in the gas-phase when liberated from A8-35 (as determined by collision cross-section (CCS) values estimated from IMS data [21]). In the following study, we describe how the physical properties of a range of APols (A8-35, A8-75, SAPol, A34-35, A34-75, and NAPol) influence the introduction of native OMPs into the gas-phase, using three OMPs: PagP, OmpT and tOmpA (Fig. 2). OmpT and PagP have been shown previously to be released from the APol A8-35 into the gas-phase in a native-like state [21], [23] but analysis of tOmpA (the transmembrane region of OmpA, residues 1–171) from A8-35 using ESI-IMS–MS is previously unreported. PagP (20.2 kDa) and tOmpA (18.9 kDa) are eight-stranded β-barrels (Fig. 2) that function as a palmitoyl transferase enzyme and porin, respectively [41], [42], [43], [44]. OmpT (35.3 kDa) is a 10-stranded β-barrel (Fig. 2) that operates as an endopeptidase, cleaving between consecutive basic residues [40], [41]. OmpT differs structurally from the other OMPs studied here, not only in that the β-barrel is larger, but also in that approximately 50% of the barrel is extra-membrane. Thus, these three OMPs provide an excellent platform for systematic investigation of the utility of different APols for stabilisation of OMPs for analysis using ESI-IMS–MS.

## 2 Materials and methods

### 2.1 OMP expression and purification

OMPs were overexpressed in BL21 (DE3) *Escherichia coli* cells in 500 mL LB culture and the bacteria harvested by centrifugation. OmpT and PagP were labelled with a His_6_ tag at the N- or C-terminus, respectively. tOmpA was expressed without an affinity tag. Cell pellets were resuspended in 50 mM Tris·HCl pH 8.0 containing 5 mM ethylenediaminetetraacetic acid (EDTA), 1 mM phenylmethanesulphonylfluoride (PMSF), 2 mM benzamidine and lysed by sonication. The lysate was pelleted by centrifugation (25,000 × *g*, 20 min, 4 °C), resuspended in 50 mM Tris·HCl pH 8.0 containing 5 mM EDTA, 2% (v/v) Triton X-100 (1 h to fully resuspend and solubilise residual membrane) and pelleted as above. Inclusion bodies were washed twice by resuspension in 50 mM Tris·HCl pH 8.0 and centrifugation as described above.

All OMPs were resuspended in 10 mM Tris·HCl pH 8.0 containing 250 mM NaCl, 6 M guanidine HCl (Gu·HCl). PagP and OmpT were initially purified by Ni^2+^-NTA affinity chromatography. Solubilised PagP or OmpT was loaded onto a 5 mL HisTrap column (GE Healthcare, Little Chalfont, Bucks, UK). The column was washed with 10 mM Tris·HCl pH 8.0 containing 250 mM NaCl, 6 M Gu·HCl, 20 mM imidazole and PagP or OmpT was eluted with 10 mM Tris·HCl pH 8.0, containing 250 mM NaCl, 6 M Gu·HCl, 250 mM imidazole.

Resolubilised tOmpA inclusion bodies and Ni^2+^-NTA-purified PagP/OmpT were purified by size exclusion chromatography (SEC). OMPs were resuspended in 10 mM Tris·HCl pH 8.0 containing 250 mM NaCl, 6 M Gu·HCl and loaded onto a Superdex 75 HiLoad 26/60 column (GE Healthcare, Little Chalfont, Bucks, UK). Protein-containing fractions were pooled and dialysed against 18 MΩ H_2_O and the precipitate was stored at −20 °C.

### 2.2 Refolding PagP/OmpT into detergent micelles

PagP or OmpT solubilised in 25 mM Tris·HCl pH 8.0 containing 6 M Gu·HCl (1 mL of 5 mg mL^−1^ OMP) was diluted dropwise into 20 mL of 10 mM Tris·HCl pH 8.0, 0.5% (w/v) *N*,*N*-dimethyldodecylamine-N-oxide (LDAO) with stirring. The OMP:LDAO solution was maintained at 4 °C overnight with mild agitation. Following syringe filtering through a 0.22 μM filter, the PagP:LDAO and OmpT:LDAO complexes were loaded onto a 1 mL HisTrap column (GE Healthcare, Little Chalfont, Bucks, UK) equilibrated with 10 mM Tris·HCl pH 8.0 containing 0.1% (w/v) LDAO. A linear gradient over 10 column volumes was used to exchange the buffer to 10 mM Tris·HCl pH 8.0, 0.02% (w/v) n-dodecyl-β-d-maltopyranoside (DDM) (n.b. 0.02% DDM is twice the detergent's CMC). OMP:DDM was then eluted using 25 mM Tris·HCl pH 8.0, 0.02% (w/v) DDM, 250 mM imidazole and immediately buffer exchanged using a Zeba™ Spin desalting column (Life Technologies Ltd., Paisley, UK) into the same buffer lacking imidazole.

### 2.3 Refolding tOmpA into detergent micelles

200 μM tOmpA in 25 mM Tris·HCl pH 8.0, 6 M Gu·HCl was diluted rapidly (20-fold) into 25 mM Tris·HCl pH 8.0, 2.9% (w/v) *n*-octyl-β-d-glucoside (β-OG) and left on ice for 10 min. tOmpA:β-OG was then incubated in a water bath at 70 °C for 3 min and returned to ice for a further 10 min, as described previously [46]. We found the most efficient protocol to create tOmpA:APol complexes was via heatshock treatment of an tOmpA:β-OG complex rather than to introduce the protein directly into A8-35 APol. Therefore, this method was used subsequently to introduce tOmpA into the different APols studied here.

### 2.4 APol trapping of OMPs

To trap OMPs in APols, each APol was added to OMP-detergent in a 1:5 (w/w) OMP:APol ratio. Following incubation on ice for 30 min, the detergent was removed by addition of BioBeads (Bio-Rad, Hemel Hempstead, Herts, UK) (20 g wet beads per g of detergent). After incubation overnight at 4 °C, samples were decanted and stored at 4 °C.

### 2.5 Cold SDS-PAGE

OMPs refolded in either detergent or APols were mixed with 2× SDS-PAGE loading buffer (50 mM Tris·HCl, pH 6.8, 2% (w/v) SDS, 0.1% (w/v) bromophenol blue and 10% (v/v) glycerol). The samples were loaded (both prior to, and immediately after, 5 min heat denaturation at 95 °C) onto a 12.5% acrylamide-Tris-Tricine SDS-PAGE gel; the gel was stained using Instant Blue stain (Expedeon Ltd., Cambridge, UK).

### 2.6 Circular dichroism

Far-UV circular dichroism (CD) spectra were acquired using a Chirascan CD spectrophotometer (Applied Photophysics, Leatherhead, Surrey, UK). An average of 5 scans between 200 and 260 nm at 20 nm min^−1^ was used. The pathlength and bandwidth were set to 0.1 mm and 1 nm, respectively. A buffer blank was used as reference and subtracted from the OMP spectra of the protein-containing sample.

### 2.7 PagP activity assay

PagP (final concentration 2.5 μM) was added to pre-filtered 25 mM Tris·HCl, pH 8.0, 1 mM p-nitrophenol palmitate (p-NPP), 10% (v/v) 2-propanol, 10% (v/v) Triton X-100. This was supplemented with 0.02% (w/v) DDM for assays of PagP activity in DDM micelles. Hydrolysis of p-NPP to p-nitrophenol (p-NP)/palmitate was monitored by observing an increase in absorbance at 410 nm [47].

### 2.8 OmpT activity assay

The self-quenching fluorogenic peptide Abz-ARRAY-NO_2_ (25 μM) (Peptide Protein Research, Hampshire, UK) was added to OmpT (125 nM) in 25 mM Tris·HCl, pH 8.0 containing 0.02% (w/v) DDM or 1:5 (w/w OmpT:APol). OmpT was incubated with 1 mg mL^−1^ lipopolysaccharide (LPS, Sigma Aldrich, Gillingham, Dorset, UK) for 1 h prior to assay. Cleavage of Abz-ARRAY-NO_2_ was monitored by observing an increase in emission at 430 nm following excitation at 325 nm using a fluorimeter (Photon Technology International, Ford, West Sussex, UK) for 300 s [21]. Specific activity was calculated for OmpT in each solubilising medium using the initial rate, endpoint fluorescence and OmpT/peptide concentration (Eq. (1)).

(1) $$\text{Specific}\;\text{activity}=\frac{\text{Initial}\;\text{rate}}{\text{Endpoint}\;\text{fluorescence}}\cdot \frac{\left[\text{substrate}\right]}{\text{OmpT}}$$

### 2.9 Mass spectrometry

All samples were buffer exchanged into 100 mM ammonium hydrogen carbonate (NH_4_HCO_3_), pH 7.8 immediately prior to ESI-IMS–MS analysis. For the DDM samples, the buffer also contained 0.02% DDM. ESI-IMS–MS experiments were conducted on a Synapt HDMS mass spectrometer (Waters Ltd., Wilmslow, Manchester, UK). OMPs were introduced into the gas-phase using a nano-ESI source and in-house manufactured gold-plated borosilicate capillaries. Capillary voltage, cone voltage, bias voltage and backing pressure were set at 1.7 kV, 70 V, 20 V, and 6 mbar, respectively. Collision voltages in the Trap (PagP and OmpT = 100–150 V; tOmpA = 50–100 V) and Transfer (50–100 V) regions prior to and immediately following the drift cell, respectively, were varied to optimise liberation of each OMP with minimal impact on its structure. The argon gas pressure in the Trap was 3.65 × 10^−2^ mbar. The IMS drift times allowed calculation of collision cross sections (CCSs) by calibration against drift times of ions of known CCS [48], [49]. Theoretical CCS values of OMPs were predicted using a scaled Projected Superposition Algorithm (PSA) from the 3D structure coordinates in the Protein Data Bank [50]. Aqueous CsI was used for *m*/*z* calibration. Data were processed using MassLynx v4.1 and Driftscope v2.5 software (Waters Ltd., Wilmslow, Manchester, UK).

### 2.10 Size exclusion chromatography to remove excess amphipol prior to ESI-MS analysis

OMP:APol samples were loaded onto an Superdex 200 10/300 GL analytical SEC column (GE Healthcare, Little Chalfont, Bucks, UK) equilibrated with 250 mM NH_4_HCO_3_, pH 7.8 (Figs. S1 and S2). OMPs were eluted with a flow rate of 0.5 ml min^−1^ and protein-containing fractions were pooled and concentrated (using a Vivaspin 2 MWCO 3,000 spin column (GE Healthcare, Little Chalfont, Bucks, UK)) prior to ESI-IMS–MS analysis.

## 3 Results and discussion

### 3.1 Refolding and subsequent exchange of PagP, OmpT and tOmpA into different APols

**Fig. 3:**
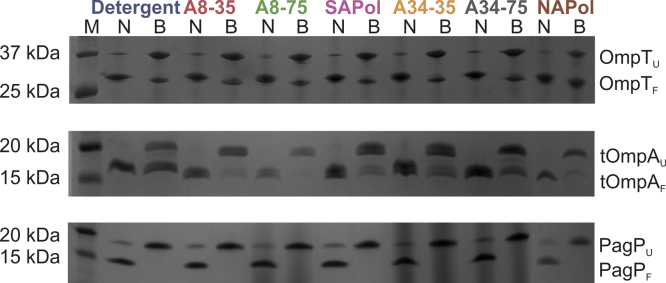
Cold SDS-PAGE indicates that OMPs refold into detergent (DDM for OmpT and PagP, β-OG for tOmpA) micelles and maintain their folding yield following exchange into APols. OMP:detergent/APol samples are loaded natively (N) or boiled (to initiate denaturation) prior to loading (B). M indicates the marker lane. Folded and unfolded conformers of OMPs are labelled as OMP_F_ and OMP_U_, respectively. The range of APols tested included A8-35, A8-75, SAPol, A34-35, A34-75, and NAPol.

**Fig. 4:**
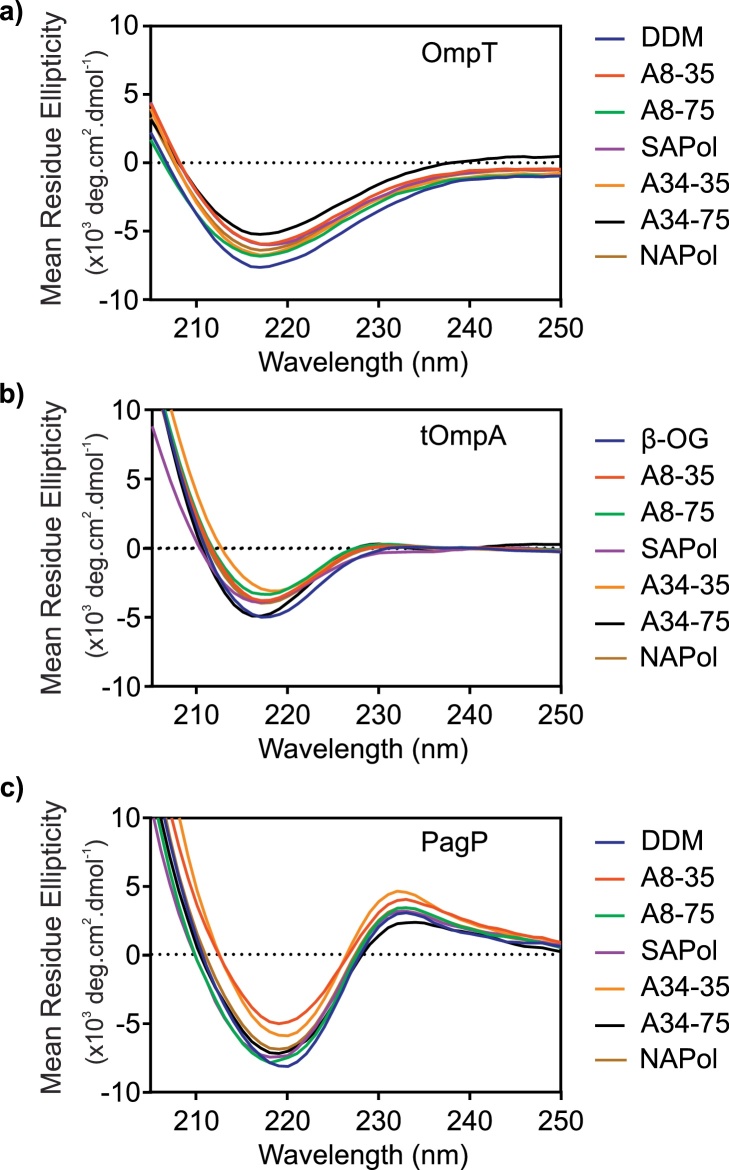
Far-UV CD spectra of (a) OmpT, (b) tOmpA and (c) PagP in different solubilising media (DDM, β-OG, or APol). The presence of the Cotton effect (maxima at 232 nm) in the spectra of PagP is characteristic of the interaction between Tyr26 and Trp66 in the protein's native state [51]. These spectra indicate that OmpT, tOmpA and PagP remain in their native states in all APols studied.

All three OMPs studied refold with high yield into detergent micelles as shown by the apparent lower molecular weight of OMPs when analysed by cold SDS-PAGE, compared with their heated counterparts (Fig. 3). Cold SDS-PAGE also indicated that folding of OMPs is maintained following exchange into each APol studied (Fig. 3). Far-UV CD experiments confirmed that the β-barrel structure expected for native OMPs is maintained following exchange into each APol (Fig. 4).

### 3.2 OmpT and PagP are catalytically active in APols

**Fig. 5:**
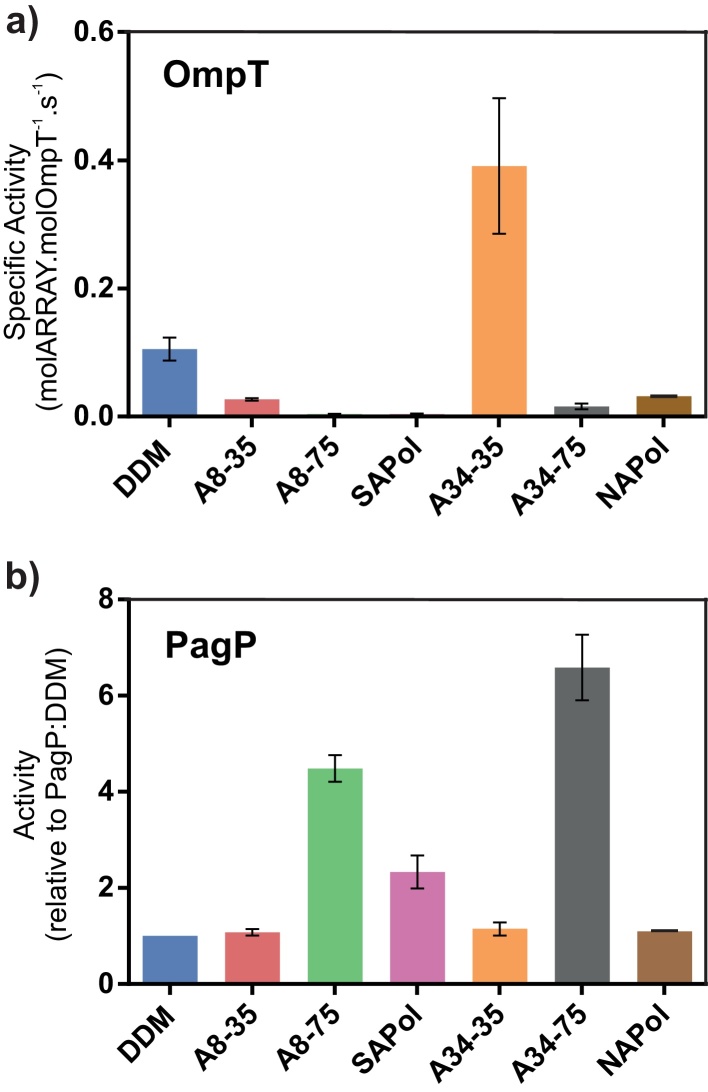
The catalytic activity of (a) OmpT and (b) PagP when solubilised by DDM or by different APols. OmpT activity is displayed as specific activity with an excess of substrate peptide and PagP activity is displayed relative to PagP:DDM activity. Error bars display standard error (*n* = 3).

To determine whether OmpT and PagP are functional in the different APols studied, the specific activity of each protein was measured and compared with the activity in DDM micelles [23], [41]. The results of these experiments showed that OmpT activity is highly dependent on the APol used to maintain solubility, despite the protein remaining in a native state in each APol studied (Fig. 5). As expected, OmpT, which is only active following prior incubation of the protein with LPS (data not shown) [23], [52], is catalytically active in DDM micelles as monitored by proteolysis of the small peptide sequence Abz-ARRAY(NO_2_) (see Section 2). Surprisingly, the enzyme is ∼4-fold more active when exchanged into A34-35, and shows decreased activity when in A8-35, A34-75, SAPol, A8-75 and NAPol (∼4, 30, 30, 7 and 3-fold decrease in specific activity, respectively, relative to OmpT in DDM micelles) (Fig. 5a). OmpT was found to be most active in the least charged APols (A8-35, A34-35 and NAPol) and have diminished activity in the more highly charged APols (A8-75, SAPol and A34-75), suggestive of an influence of surfactant charge on its activity. Cold SDS-PAGE (Fig. 3) and CD data (Fig. 4) suggest that any structural perturbations that may result in this change in activity are subtle and not detectable by these methods. OmpT:A8-35 activity (0.87 μmoles Abz-ARRAY(NO_2_) molOmpT^−1^ s^−1^) is highly comparable to that observed previously in A8-35 [21].

The PagP activity assay monitors the hydrolysis of p-nitrophenolpalmitate (p-NPP) (a palmitoyl substrate) to p-nitrophenol (p-NP). PagP activity appears to have an inversed (and more subtle) sensitivity to the charge of the APol used compared with OmpT. PagP shows similar activity in DDM micelles, A8-35, A34-35 and NAPol, but an increase in activity in SAPol, A8-75 and A34-75 (Fig. 5b). This, as in the case of OmpT, suggests that the density of charges carried by APols may cause subtle, local structural perturbations that affect the catalytic efficiency of PagP.

### 3.3 Gas-phase liberation of OMPs from APols

To determine whether the different APols alter the efficiency of release of the OMPs into the gas-phase for ESI-MS analysis, OmpT, tOmpA and PagP, solubilised independently in detergent micelles and in each APol, were introduced into the mass spectrometer using nano-ESI. Their charge state distributions and collision cross-sectional areas (CCS) were then used to investigate the structural properties of each protein in the gas-phase. OMP:DDM or OMP:APol complexes were subjected to collisional-activation in the Trap region prior to the IMS drift cell of the mass spectrometer in order to release the OMP from the amphiphile [21], [23]. The process of OMP liberation from amphiphile was optimised by maintaining ESI sampling cone, Trap and Transfer (the region following the IMS drift cell) voltages at the lowest values possible whilst ensuring satisfactory release of protein from each amphiphile.

**Fig. 6:**
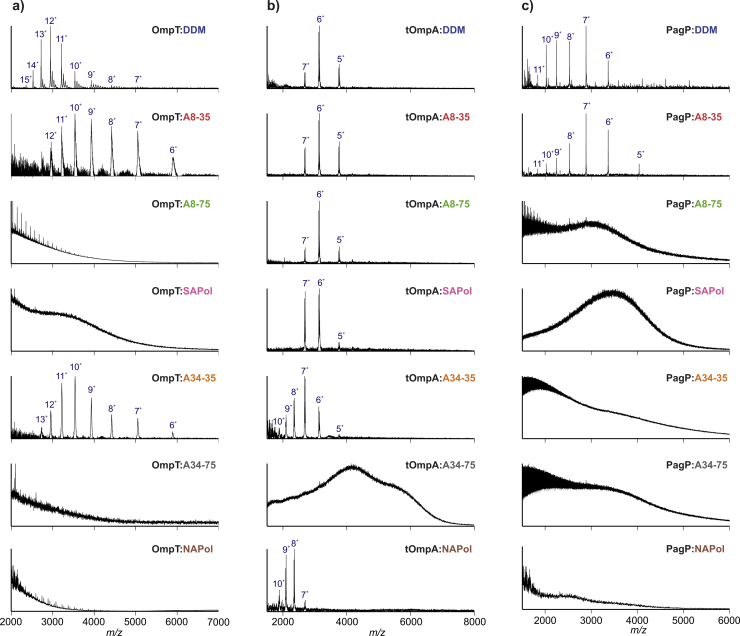
ESI-MS spectra of (a) OmpT, (b) tOmpA and (c) PagP solubilised in DDM micelles or APol (A8-35, A8-75, SAPol, A34-35, A34-75 or NAPol), as indicated. The tOmpA:A34-35 spectrum was acquired following purification by SEC. All other spectra shown were obtained without prior SEC.

Liberation of OMPs (from APols or DDM micelles) was found to be highly dependent on which medium is used to solubilise the protein. All OMPs were released readily from DDM micelles and A8-35. For tOmpA and PagP, in each case the spectra acquired from DDM micelles and A8-35 are comparable, showing a similar distribution of charge states i.e. 5^+^ to 7^+^ and 5^+^ to 11^+^ for tOmpA and PagP, respectively (Fig. 6). The charge state distribution of OmpT differs when liberated from detergent (7^+^ to 15^+^) or A8-35 (6^+^ to 13^+^), the higher charge states of the detergent:OmpT complex indicating a more expanded species (Fig. 6). OmpT also exhibits DDM adducting. Of the three OMPs studied under these conditions, this difference in charge state distribution is only observed for OmpT. OmpT has approximately 50% of its β-barrel protruding from a native lipid bilayer in vivo (Fig. 2); hence, it is feasible that APols could interact with this extra-membrane surface and prevent extra charging of OmpT or, a degree of unfolding of this exposed region caused by the dissociating nature of DDM [14], [53], or by collisional activation of the protein in DDM, could occur.

Release from the other APols is variable among the OMPs (Fig. 6). OmpT is released readily from A34-35 (the medium in which OmpT has the greatest catalytic activity), but not from the more highly negatively charged APols, i.e. A8-75, SAPol or A34-75, nor from NAPol. PagP also resisted liberation from the more highly negatively charged APols (A8-75, SAPol and A34-75) (in which PagP has greater activity than in DDM micelles or in less negatively charged APols), as well as from A34-35 and NAPol. tOmpA exhibited more ubiquitous behaviour, being released from A8-35, as well as the more highly negatively charged APols A8-75 and SAPol, and also from NAPol but not from A34-75. tOmpA was also liberated successfully from A34-35, but only following size-exclusion chromatography (SEC) separation of the protein from excess APol. Interestingly, following SEC, the charge state distribution of ions of tOmpA liberated from A34-35 was extended to include higher charge states (i.e. 5^+^ to 10^+^ compared with 5^+^ to 7^+^ for analysis from most of the other APols without a SEC purification step) (Fig. 6). The tOmpA spectrum acquired following release from NAPol also contained ions with higher charge states (7^+^ to 10^+^), although the SEC purification step was not necessary in this case. Data acquired from the other two OMPs from APols subsequent to removal of excess APol by SEC showed similar extensions of the charge state distributions (Fig. S1). A likely explanation for this is the possible greater exposure of ionisable regions of the proteins when excess APol, which may or may not have been bound to the OMP, is removed from the mixture. As the OMPs are still in an active form after this treatment, it is unlikely that any additional ionisation is due to wide-scale protein unfolding. This is supported by our ESI-IMS–MS studies which indicate that the majority of the Omp ions populate a compact state in the gas-phase with only a trace of a lowly populated, expanded conformer observed for the most highly charged ions (see Section 3.4). Although the SEC enrichment step enabled the generation of mass spectra from tOmpA in A34-35 when otherwise spectra could not be obtained, it did not prove successful for the analysis of tOmpA from A34-75, nor for the other unsuccessful analyses reported in Fig. 6 (data not shown).

Taken together, the results show that of the APols studied, A8-35 is the most universal, as all three OMPs could be analysed routinely from this medium using ESI-MS. Its higher mass counterpart, A34-35, was successful for the analysis of two of the three OMPs (OmpT and tOmpA). The more highly negatively charged APols did not perform as well: none of the OMPs was detected when analysed from A34-75, and only tOmpA was analysed successfully from A8-75, SAPol or from the non-ionic Apol, NAPol.

The results indicate that APols which are large and/or highly charged are limited in their power to deliver OMPs to the gas-phase. With respect to the influence of APol size, this could result from the high kinetic stability of OMP:APol complexes [54] (arising from the large number of contacts made between an OMP and a single APol molecule). OmpT (which is released from A34-35 without any prior SEC purification from excess APol) may be less resistant to liberation, as a result of the exposed β-barrel surface that is absent in tOmpA and PagP.

The inconsistent release of OMPs from the more highly negatively charged A8-75 and SAPol, and the negative results with A34-75, indicate that charge is impacting the observation of OMPs in the gas-phase. Most APols are negatively charged species, a single molecule of A34-75 having a net charge of ca. −110 (assuming that it was derived from a polyacrylamide precursor of average mass 16 kDa, has 75% of acid groups which remain ungrafted and that 100% of free acids are deprotonated and lacking counterions) and could well be difficult to ionise using positive mode ESI (although none of the OMP, APol or OMP:APol complexes could be observed using negative mode ESI (data not shown)). With the exception of the small membrane-embedded helix of PagP, there is no obvious structural or physical difference that would suggest why the behaviour of the OMP:APol complexes in their ionisation efficiencies differ so significantly.

### 3.4 ESI-IMS–MS analysis of liberated OMPs

**Fig. 7:**
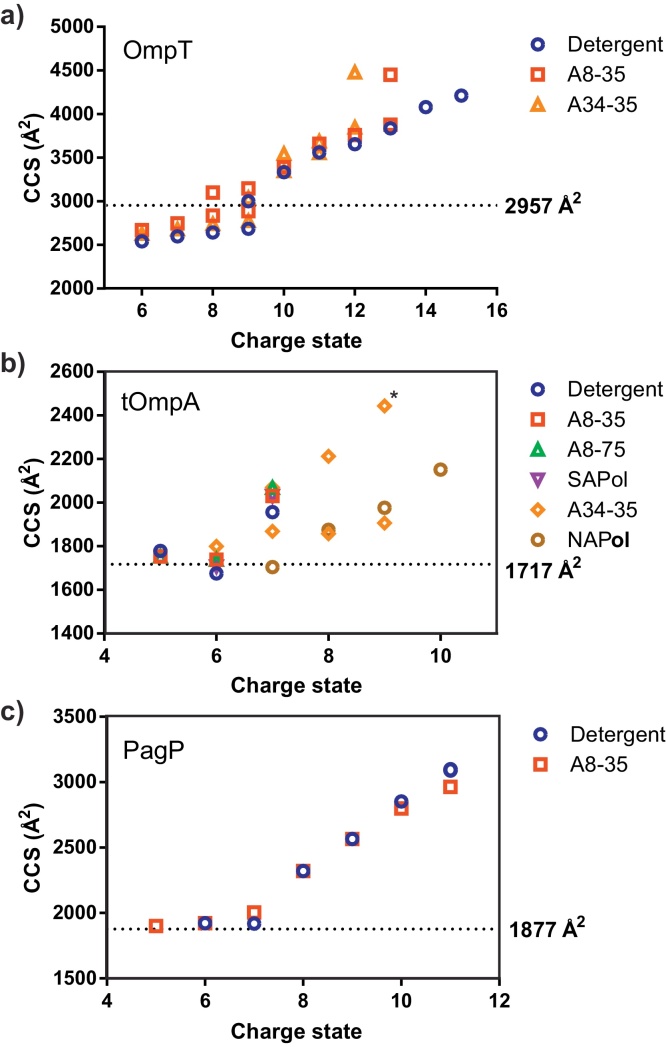
CCS-charge relationships for (a) OmpT, (b) tOmpA and (c) PagP ions released from DDM micelles or APols. The tOmpA:A34-35 spectrum was acquired following purification by SEC. CCS values were estimated using ESI-IMS–MS data acquired under identical instrumental conditions (see Section 2). * in (b) indicates low intensity ions difficult to measure CCS accurately. Dashed lines indicate the value of the theoretical OMP CCS calculated from the PDB co-ordinates of crystal structures of each protein [42], [44], [45] using a PSA algorithm [50].

ESI-IMS–MS analysis of OMPs indicates that the lowest charge state ions of all proteins investigated adopt a compact conformation, with CCS values that compare quite favourably with the predicted values from the crystal structures of each protein (i.e. OmpT (2957 Å^2^), tOmpA (1717 Å^2^) and PagP (1877 Å^2^)), Fig. 7 and Fig. S3. An increase in charge state is associated with protein unfolding and hence more expanded OMP ions. Where OMP ions are observed, CCS-*z* relationships are comparable irrespective of the solubilising media used. For example, the highly populated, lowest charge state ions (5^+^ to 7^+^) of tOmpA released from the SEC-purified complex with A34-35 populate the same compact conformational profile as tOmpA released from the other APols or from detergent, although a small proportion of ions that occupy the higher charge states (8^+^, 9^+^) populate a more extended conformer are observed in the presence of A34-35 following SEC purification which are not seen in the presence of other APols or DDM (Fig. 7b). These data are consistent with observations by CD and cold SDS-PAGE which show that APols conserve the global structure of OMPs (Fig. 3, Fig. 4) and also confirm the fact that native-like conformers of OMPs liberated from APols can persist in the gas-phase.

For OmpT, regardless of the amphiphile used for the ESI-MS analysis, the most compact conformer ions occupy the 6^+^ to 9^+^ charge states with an estimated CCS of ca. 2600 Å^2^, i.e. ca. 10% more compact than the CCS calculated form the protein's PDB co-ordinates (2957 Å^2^) (Fig. 7 and Fig. S3). At least one more extended Omp T conformer is detected for the more highly charged ions (8^+^ to 13^+^) indicating a degree of unfolding. The expected error associated with travelling wave ESI-IMS–MS CCS experiments is in the region of 5–7%, so the observed CCS for the most compact OmpT conformer is smaller than may be expected. However, this phenomenon has been reported elsewhere for OmpT and a conformational collapse in the gas-phase, likely in the loop regions, has been proposed as an explanation [23]. Similarly, OmpA analysis using ESI-IMS–MS generated CCS values lower than predicted from the corresponding crystal structure and this gas-phase collapse was accounted for also by the presence of the flexible loops on the extra-cellular side of the MP [55].

PagP shows a similar conformeric profile: the 5^+^ to 7^+^ ions (ca. 1900 Å^2^) are consistent with the calculated CCS of the native protein (1877 Å^2^). The more highly charged state ions (8^+^ to 11^+^) occupy a more extended conformation (CCS values > 2300 Å^2^), as may be expected due to Coulombic repulsion. Again, under these conditions, the conformeric profiles of the protein are similar, regardless of whether APol or DDM is used for the analysis. tOmpA has a similar conformeric pattern, with the 5^+^ and 6^+^ charge state ions having an estimated CCS of ca. 1700 Å^2^ (compared with 1717 Å^2^ predicted from the PDB co-ordinates), and the higher charge states showing larger CCS (>1900 Å^2^) (Fig. 7 and Fig. S3). However, in the case of tOmpA, the majority of the protein occupies the most compact conformer. For each OMP studied, the CCSs of the lowest charge states are consistent with the protein occupying a compact state. PagP and OmpT occupy their respective higher charge state ions to a greater extent when analysed from DDM compared to when analysed from the APol A8-35, indicating a more stable, native-like protein in the presence of the APol.

## 4 Conclusion

Here we have shown that OMP structure can be preserved efficiently in a variety of APols by introduction of the protein into the APol via different detergents, maintaining their native structure and, where appropriate, enzyme activity. The specific properties of the different APols studied appear to elicit protein-specific effects on the activity of OMPs, possibly through local structural perturbations too subtle to be observed using cold SDS-PAGE, CD or ESI-IMS–MS and hence that do not influence the global OMP structure. DDM micelles and A8-35 APol are shown here to be the most reliable of the above tested amphiphiles for delivering native-like OMPs into the gas-phase in a compact conformation. Increasing the size or negative charge of the APols has been found to be detrimental when trying to observe free OMPs in the gas-phase. Regardless of the APol used, OMPs released into the gas-phase retain compact, native-like structures for their lowest charged ions, with CCSs in agreement with PDB co-ordinates. The results also show that the OMP and APol mixtures need to be optimised for each complex studied, and highlight the utility of APols, especially A8-35, for maintaining OMPs in a stable, native-like conformation for ESI-IMS–MS and other biophysical analyses.
