# Supplementary material for: Systematic analysis of the use of amphipathic polymers for studies of outer membrane proteins using mass spectrometry
Source: Int J Mass Spectrom. 2015 Nov 30;391:54–61. doi: 10.1016/j.ijms.2015.06.017 (PMC4708066; doi:10.1016/j.ijms.2015.06.017)
Supplement: Supplementary file 1 [file mmc1.pdf]

Systematic analysis of the use of amphipathic polymers for studies of outer membrane proteins using mass spectrometry

Thomas G. Watkinson[1], Antonio N. Calabrese[1], Fabrice Giusti[2], Manuela Zoonens[2], Sheena E. Radford[1*], Alison E. Ashcroft[1*]

[1]Astbury Centre for Structural Molecular Biology, School of Molecular & Cellular Biology, University of Leeds, Leeds, LS2 9JT, UK

[2]Laboratoire de Physico-Chimie Moléculaire des Protéines Membranaires, UMR 7099, Institut de Biologie Physico-Chimique (FRC 550), Centre National de la Recherche Scientifique/Université Paris-7, 13, rue Pierre-et-Marie-Curie, 75005 Paris, France

Supplementary Material

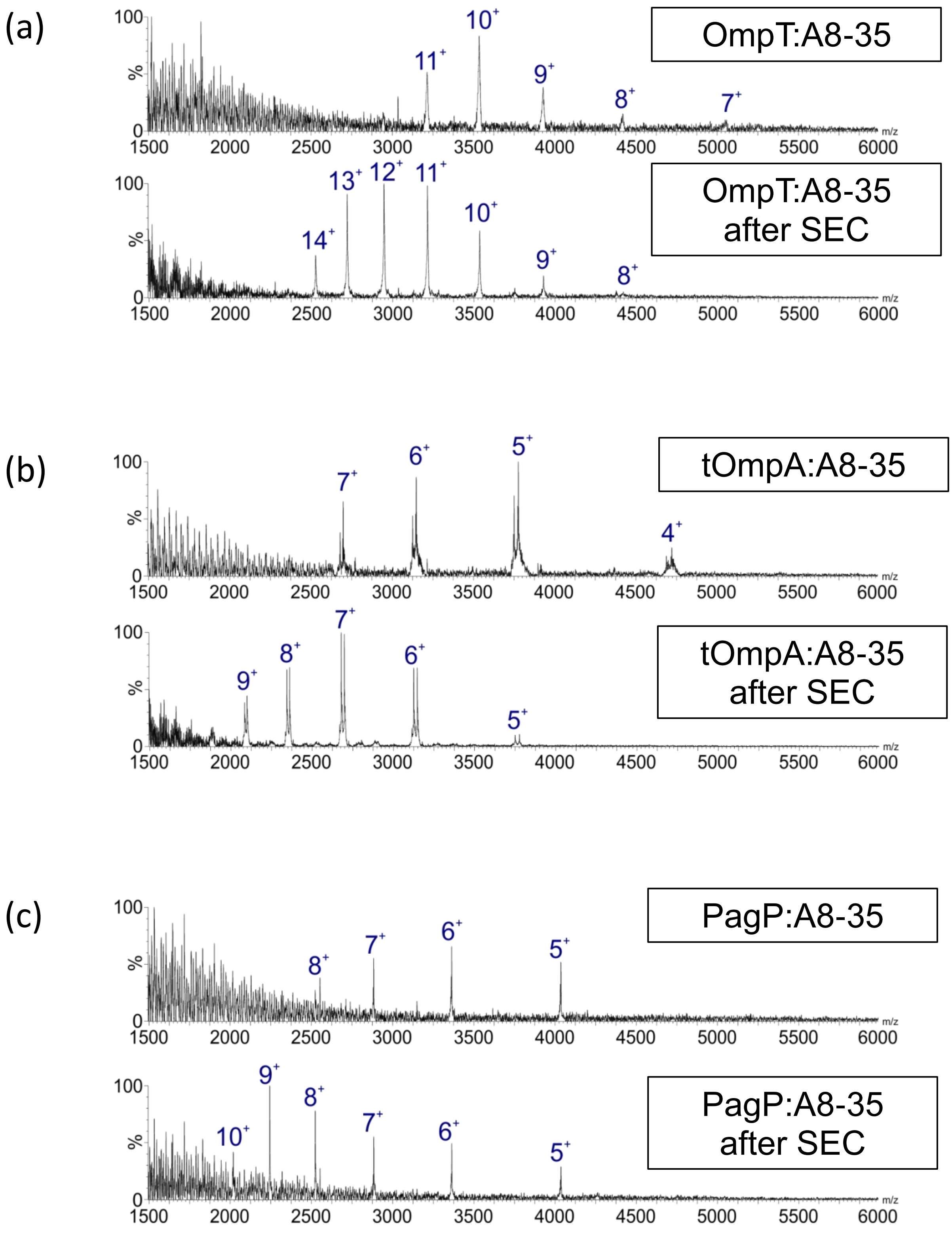

Figure S1. OmpT, tOmpA and PagP were exchanged into A8-35 APol and purified by SEC (Superdex 200 analytical column equilibrated in 250 mM ammonium bicarbonate, pH 7.8). The protein-containing fractions were analysed by ESI-MS. A comparison of each OMP with and without SEC purification is shown for (a) OmpT, (b) tOmpA, and (c) PagP. Released OMPs have a higher charge state distribution following SEC than their precursor counterparts. N.b. the presence of two peaks for each charge state in (b) is due to the presence of the protein with and without an N-terminal methionine residue.

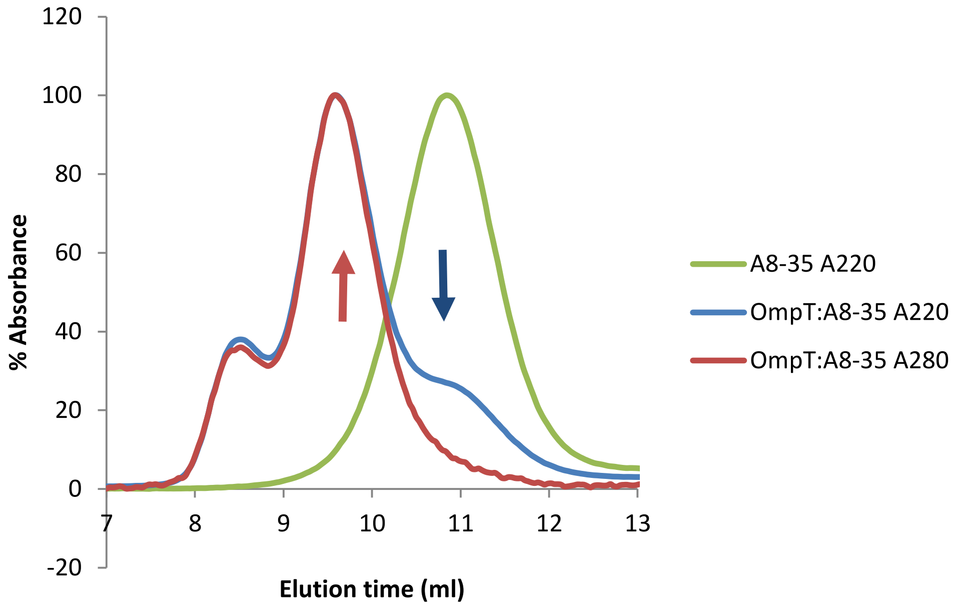

Figure S2. SEC separation of OmpT:A8-35 from excess A8-35. A8-35 absorbs at 220 nm but not 280 nm. The OmpT:A8-35 peak (detected at 220 (blue trace) and 280 (red trace) nm) is separated from excess, free A8-35 (220 nm, green trace). The OmpT:A8-35 220 nm peaks (blue trace) without a corresponding 280 nm signal (i.e. as indicated by the downward facing blue arrow) are a result of free A8-35. The OMP:APol complex can be isolated from free A8-35 by pooling fractions from the peak identified by the upward facing red arrow.

Protein<br>calculated CCS<br>PDB	z	CCS (A^2)					
		Detergent	A8-35	A8-75	SAPol	A34-35	NAPol
OmpT<br>2957 $Å^2$<br>PDB: 1I78[1]	6	2541	2671	-	-	2629	-
	7	2599	2748	-	-	2681	-
	8	2643	2839	-	-	2741	-
	8	-	3103	-	-	-	-
	9	2685	2888	-	-	2777	-
	9	3004	3151	-	-	3042	-
	10	3339	3397	-	-	3351	-
	10	-	-	-	-	3551	-
	11	3567	3666	-	-	3559	-
	11	-	-	-	-	3693	-
	12	3660	3766	-	-	3854	-
	12	-	-	-	-	4490	-
	13	3843	3886	-	-	-	-
	13	-	4460	-	-	-	-
	14	4089	-	-	-	-	-
	15	4221	-	-	-	-	-
PagP<br>1877 $Å^2$<br>PDB:<br>1THQ[2]	5	-	1901	-	-	-	-
	6	1923	1923	-	-	-	-
	7	1919	2004	-	-	-	-
	8	2321	2321	-	-	-	-
	9	2566	2566	-	-	-	-
	10	2852	2800	-	-	-	-
	11	3081	2964	-	-	-	-
tOmpA<br>1717 $Å^2$<br>PDB: 1QJP[3]	5	1779	1753	1753	-	-	-
	6	1676	1739	1739	1707	1799	-
	7	1957	2031	2067	2031	1869	1704
	7	-	-	-	-	2070	-
	8	-	-	-	-	1858	1876
	8	-	-	-	-	2213	-
	9	-	-	-	-	1907	1977
	9	-	-	-	-	2443*	-
	10	-	-	-	-	-	2152

Figure S3. Rotationally-averaged collision cross-sectional (CCS) values determined for OMP proteins analysed by ESI-IMS-MS from their respective solubilising media. Theoretical CCS values and PDB codes for respective source structures are indicated in far left column. * indicates low intensity ions difficult to measure CCS accurately. n.b. The tOmpA:A34-35 data were acquired following purification by SEC.

1. L. Vandeputte-Rutten, R. A. Kramer, J. Kroon, N. Dekker, M. R. Egmond, P. Gros, EMBO J. 20 (2001) 5033-5039.

2. V. E. Ahn, E. I. Lo, C. K. Engel, L. Chen, P. M. Hwang, PL. E. Kay, R. E. Bishop, G. G. Privé, EMBO J. 23 (2004) 2931-2941.

3. A. Pautsch, G. E. Schulz, J. Mol. Biol. 298 (2000) 273-282.
